# Clinically Silent Small Vessel Disease of the Brain in Patients with Obstructive Sleep Apnea Hypopnea Syndrome

**DOI:** 10.3390/diagnostics11091673

**Published:** 2021-09-13

**Authors:** Dimitrios G. Raptis, Olga Sinani, Georgia G. Rapti, Aikaterini Papanikolaou, Katerina Dadouli, Panagiotis Ntellas, Eftychia Z. Kapsalaki, Foteini Malli, Konstantinos I. Gourgoulianis, Georgia Xiromerisiou

**Affiliations:** 1 Respiratory Medicine Department, School of Medicine, University of Thessaly, 41110 Larissa, Greece; raptisdmed@gmail.com (D.G.R.); oliarapti@gmail.com (G.G.R.); kgourg@med.uth.gr (K.I.G.); 2 Faculty of Medicine, School of Health Sciences, University of Thessaly, 41222 Larissa, Greece; oliasin@hotmail.com (O.S.); katerina_papanikolaou@hotmail.com (A.P.); 3 Laboratory of Hygiene and Epidemiology, Faculty of Medicine, University of Thessaly, 41222 Larissa, Greece; katerina1dad@gmail.com; 4 Department of Medical Oncology, University Hospital of Ioannina, 45500 Ioannina, Greece; ntellasp@gmail.com; 5 Department of Diagnostic Radiology, Faculty of Medicine, General University Hospital of Larissa, University of Thessaly, 41110 Larissa, Greece; ekapsal@med.uth.gr; 6 Respiratory Disorders Lab, Faculty of Nursing, University of Thessaly, 41500 Larissa, Greece; 7 Department of Neurology, School of Medicine, University of Thessaly, 41110 Larissa, Greece; georgiaxiromerisiou@gmail.com

**Keywords:** obstructive sleep apnea hypopnea syndrome, small vessel disease, white matter

## Abstract

Obstructive sleep apnea hypopnea syndrome (OSAHS) is associated with increased risk of cerebrovascular disease. The aim of the present study was to investigate the association between the presence of the small vessel disease (SVD) of the brain in patients with OSAHS. The study included 24 patients with moderate to severe OSAHS and 34 healthy volunteers. All the subjects underwent magnetic resonance imaging (MRI) of the brain, in order to sought periventricular white matter (PVWM), deep white matter (DWM) and brainstem SVD. Among patients with OSAHS, 79.1% had SVD (grade 1–3, Fazekas score) in DWM and 91.7% in PVWM while 22.4% had brainstem—white matter hyperintensities (B-WMH). Patients with OSAHS had a much higher degree of SVD in the DWM and PVWM compared to the control group (*p* < 0.001). The multivariate analysis showed an independent significant association of OSAHS with SVD (DWM and PVWM) (*p* = 0.033, OR 95% CI: 8.66 (1.19–63.08) and: *p* = 0.002, OR 95% CI: 104.98 (5.15–2141)). The same analysis showed a moderate association of OSAHS with B-WMH (*p* = 0.050, OR 15.07 (0.97–234.65)). Our study demonstrated an independent significant association of OSAHS with SVD and a moderate association of OSAHS with B-WMH.

## 1. Introduction

Obstructive sleep apnea hypopnea syndrome (OSAHS) affects 5–10% of the population. The severity of the syndrome in determined by the number of apneas and hypopneas taking place during an hour of sleep (apnea-hypopnea index—AHI) as well as by the seriousness of the patient’s symptoms at daytime [1,2]. The pathophysiology of OSAHS is multifactorial and possibly lies on a genetic background, as well as on various factors, such as obesity that may contribute significantly [1].

OSAHS impact on patients with ischemic stroke (IS) or transient ischemic attack is quite higher when compared to the general public. It also seems that OSAHS constitutes a factor that influences the course of an IS and the patient’s functionality, after the stroke [3,4]. Moderate-to-severe OSA is more frequent in wake-up strokes compared to non-wake up strokes [5]. A recent study observed a high prevalence and variability of Sleep-disordered breathing in the acute phase of cerebral ischemic events [6]. However, the correlation between OSAHS and cerebrovascular disease has not been studied extensively and a lot of queries remain unanswered, such as, the pathophysiological mechanism that links them. It seems that the pathophysiological cataract caused by OSAHS, includes oxidative stress, inflammation, sympathetic system activation, endothelial dysfunction and, finally, metabolic derangement (resistance to insulin and lipid derangement), which lead to damage of microvessels in the brain [7].

Small vessel disease (SVD) of the brain result from the damage taking place on penetrating cerebral microvessels, capillaries and veins, that damage the underlying tissue, the white and grey matter of the brain. Penetrating vessels play a crucial role in retaining the functionality of the brain and especially of nuclei with high metabolic needs, as well as the complex networks of the white matter [8]. The detection of microvascular brain disease is important due to its association with clinically significant IS and its possible contribution to cognitive impairment. The association of OSAHS with microvascular brain damage has not been extensively studied in the current literature.

The objective of the present study was to investigate the association between the presence of SVD in patients with OSAHS in a Greek population. Furthermore, we aim to evaluate the impact of OSAHS in brainstem—white matter hyperintensities (B-WMH) since little is known regarding B-WMH topography.

## 2. Materials and Methods

### 2.1. Study Design and Participants

**Figure 1:**
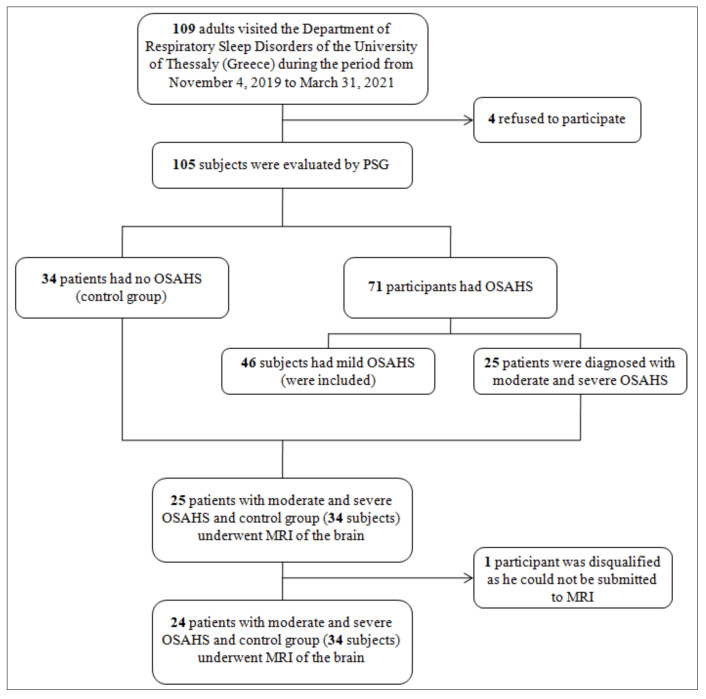
Flowchart of the study selection process and research workflow. List of Abbreviations: OSAHS: Obstructive sleep apnea hypopnea syndrome, PSG: Polysomnography, MRI: Magnetic resonance imaging.

A total of 109 adults were approached at their appointment at the Department of Respiratory Sleep Disorders of the University of Thessaly (Greece) during the period from 4 November 2019 to 31 March 2021 (Figure 1). All patients were informed about the study and four refused to participate. All participants gave written consent. The 105 participants were evaluated for the possible existence of OSAHS by standard polysomnography (PSG) according to the American Academy of Sleep Medicine (AASM) guidelines [1] for the diagnosis of OSAHS. Forty-six participants had mild OSAHS (5 ≤ AHI ≤ 15) so they were not included in the study. Thirty-four patients did not have OSAHS (AHI < 5) and were the control group of the study. The remaining 25 participants were diagnosed with moderate and severe OSAHS (15 < AHI ≤ 30 and AHI > 30, respectively). The 24 participants (one participant was disqualified as he could not be submitted to MRI) with moderate and severe OSAHS and the control group underwent brain MRI to assess the presence of cerebral SVD. Specifically, we searched for vascular lesions in the periventricular white matter (PVWM), deep white matter (DWM), midbrain, pons and medulla oblongata. All demographic characteristics and somatometric data were recorded, a complete individual history was obtained and a complete clinical examination was performed. Hypertension was defined as systolic blood pressure values ≥ 140 mmHg and/or diastolic blood pressure values ≥ 90 mmHg [9]. Coronary artery disease (or ischemic heart disease) was defined as the state of inadequate supply of blood to the myocardium due to obstruction of the epicardial coronary arteries, usually from atherosclerosis [10] Atrial fibrillation (AF) was defined as a supraventricular tachyarrhythmia during which atrium is contracted ineffectively with electrocardiographic characteristics of irregular R-R intervals, absence of distinct repeating P waves, and irregular atrial activations [11]. The definition of dyslipidemia included elevated levels of low-density lipoprotein (LDL) or total cholesterol and/or decreased levels of high-density lipoprotein cholesterol. More specifically, the condition is diagnosed in the presence of elevated total cholesterol > 200 mg/dL or low-density lipoprotein (LDL) > 100 mg/dL, or low levels of high-density lipoprotein (HDL) < 50 mg/dL [12]. Peripheral arterial disease was defined as atherosclerotic disease leading to peripheral artery obstruction [13]. Diabetes mellitus refers to a total of metabolic diseases with increased levels of glucose in the blood plasma, as a result of defective insulin secretion, insulin action, or both [14].

### 2.2. Ethics

Patients and controls provided written informed consent. The study was approved by the Independent Ethical Committee of the University of Thessaly, Medical School (No. 13—12/02/2019).

### 2.3. Polysomnography

The participants of the study underwent polysomnography. During this procedure, electroencephalography, electrooculography, electromyography, electrocardiography and oronasalthermistor were used. Bonds were attached to the thoracic cage and the abdomen in order to monitor the respiratory motions. Oxygen was monitored with a pulse oximeter. Apnea was defined as a ≥90% reduction in airflow lasting at least 10 s. Hypopnea was defined as a ≥30% reduction in airflow lasting at least 10 s and at least a 3% drop (or arousal) in oxygen saturation. The AHI score was calculated as the average number of apneas and hypopneas per hour of sleep. According to the AASM [1] OSAHS was classifies based on the AHI as mild (5 ≤ AHI ≤ 15), moderate (15 < AHI ≤ 30) and severe (AHI > 30).

### 2.4. MRI Brain Protocol and Assessment

MRI was performed in all participants using a standard protocol. A 3-T Magnetic Resonance Scanner (GE HDx, Milwaukee, WI, USA) was used and the protocol included whole-brain T2-weighted, T1-weighted, and T2*-weighted gradient-recalled echo, FLAIR, as well as diffusion sequences. The assessment of the MRI images was performed by a neuroradiologist that was blinded to the participants’ clinical data.

White matter lesions were defined as follows: Any signal abnormality despite of size that is located in the white matter displaying hyperintensity on T2-weighted images like fluid-attenuated inversion recovery, that does not present cavitation [15].

We used the Fazekas score (FS) to evaluate WMH on axial T2-weighted and FLAIR sequences [16]. Patients were classified according to FS to the following stages: 0 (no lesions), 1 (nonconfluent lesions), 2 (confluent lesions), and 3 (diffuse lesions). We evaluated cerebral microbleeds on T2*-weighted gradient sequences with a dichotomized classification scheme as present or absent.

Additionally, we assessed for B-WMH. We defined B-WMH as areas of hyperintensity without the presence of distinct borders on FLAIR images, presenting with minor or absent corresponding hypointensity on T1 -weighted images in mesencephalon, pons, and/or medulla. The lesions were classified by a neuro -radiologist (SM) with a dichotomized classification scheme as absent or present. Lesions with clear demarcated hyperintensity located in the brainstem that were associated with a corresponding DWI hyperintensity or clear T1-weighted hypointensity were considered infarcts and were not included in the analysis. Since the WMH areas located in the brainstem are too small, we cannot adequately discriminate B-WMH according to volume by the semiautomated volumetric image analysis as we performed to PV (periventricular) and D (deep) WMH. The clinical data were not available to the readers of the MRI images. We used the Microbleed Anatomical Rating Scale to identify deep microbleeds [17].

### 2.5. Statistical Analysis

Continuous variables are expressed as means ± standard deviations and categorical variables as frequencies and proportions. Student’s *t*-Test was performed for continuous data since there were no deviation from normal distribution (Shapiro-Wilk normality test) and violation of assumption of homogeneity of variance (Levene’s test). Categorical variables were compared using the χ2test.Multivariate analysis was performed in the form of binary and multinomial logistic regression. In multivariate analysis, factors with a *p* value < 0.10 in univariate analysis included. For all the analyses a 5% significance level was set. Analyses were performed using SPSS version 22 for Windows^®^ (Chicago, IL, USA).

## 3. Results

The patients with OSAHS consisted of 24 patients (males: 62.5%) with a mean age of 58 ± 11.9 years. Of the 24 patients with OSAHS, 16 had severe syndrome and 8 had moderate. The mean AHI was 40.4 ± 21.2 events/h. Mean apnea index (AI) was 16.3 ± 17.3 events/h and mean hypopnea index (HI) was 24.1 ± 13.4 events/h. Oxygen desaturation index (ODI) was 41.2 ± 23.2 events/h, time with hemoglobin saturation < 90% (T < 90%) was 36.8 ± 65.6 min and that of the minimum SaO_2_ level was 76.5 ± 12.8%. Sleep parameters and characteristics of OSAHS patients are presented in Table 1. The control group consisted of 34 participants (males: 58.82%) with a mean age of 57.6 ± 10.8 years. Vascular comorbidity of OSAHS patients and controls are presented in Table 2. Patients with OSAHS had hypertension in 50%, elevated blood cholesterol levels in 58.3% and 41.7% were smokers. Diabetes was present in 16.7% of the patients. None of the patients had a thyroid condition. Patients with hypertension received angiotensin-converting enzyme (ACE) inhibitors (ramipril, enalapril). Patients with coronary artery disease were under statin therapy, antiplatelets, ACE inhibitors and b-blockers. Atrial fibrillation patients, as well as patients with pulmonary embolism received direct-oral anticoagulants (DOACs). Dyslipidemia was treated with statins. Stroke patients received antiplatelets and DOACs and peripheral arterial disease was treated with antiplatelet agents. All the diabetics patients that we included in the study had type 2 diabetes and they did not present any symptoms of peripheral or central neuropathy. All the diabetic patients were receiving metformin as the main antidiabetic medication.

### MRI Results

**Figure 2:**
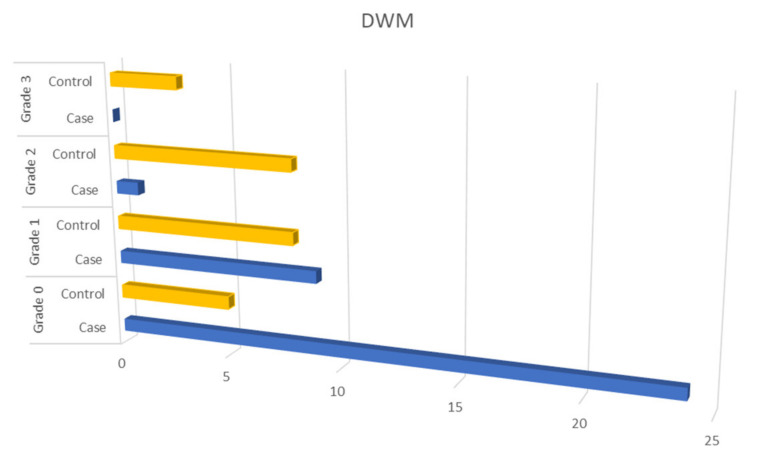
Comparison of patients with OSAHS and controls group with respect to SVD in the DWM.

**Figure 3:**
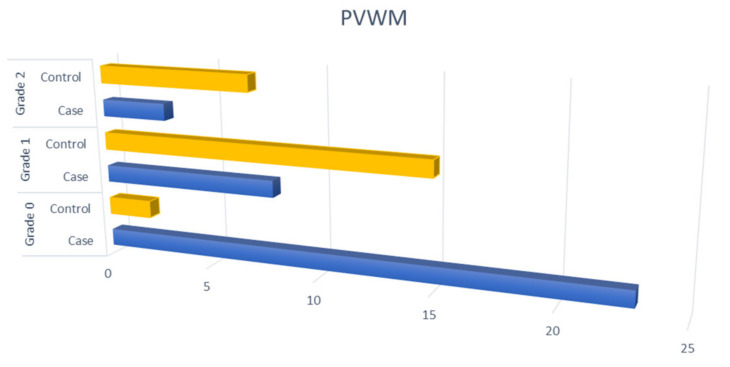
Comparison of patients with OSAHS and control group with respect to SVD in the PVWM.

**Figure 4:**
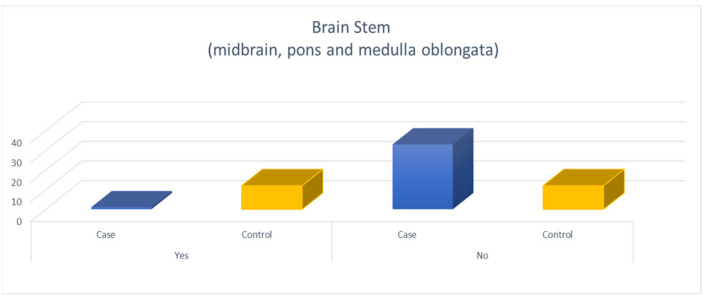
Comparison of patients with OSAHS and the control group for the presence of B-WMH.

Among patients with OSAHS, 79.1% had SVD (grade 1–3, Fazekas score) in DWM and 91.7% in PVWM while 22.4% had B-WMH. Patients with OSAHS had a much higher degree of SVD in the DWM, PVWM compared to the control group with statistically significant difference (*p* < 0.001) (Figure 2 and Figure 3). When comparing patients with OSAHS and the control group for the presence of B-WMH, patients with OSAHS showed statistically significant more lesions (*p* < 0.001) (OR: 33 (3.87–281.70)) (Figure 4).

According to the univariate analysis, the main factors that affect the presence of SVD in the deep white matter are sex with males being more affected than women (*p* = 0.097), coronary artery disease (*p* < 0.001), hypertension (*p* < 0.001) and diabetes (*p* = 0.039). The duration of the disorder (OSAHS) is also an important factor that affects the presence of SVD (*p* < 0.001) (Figure S1a–d)).

The main factors that affect the presence of SVD in the periventricular white matter, according to the univariate analysis are sex, with males being much more affected than women (*p* = 0.035), coronary artery disease (*p* = 0.017) and hypertension (*p* = 0.002). The duration of the disorder (OSAHS) is also an important factor that affects the presence of SVD (*p* < 0.001) (Figure S2a–c).

The multivariate analysis showed an independent significant association of OSAHS with SVD (DWM and PVWM) (*p* = 0.033, OR 95% CI: 8.66 (1.19–63.08) and *p* = 0.002, OR 95% CI: 104.98 (5.15–2141), respectively) (Table 3). The same analysis showed a marginally not statistically significant association of OSAHS with B-WMH (*p* = 0.053, OR 15.07 (0.97–234.65)) (Table 3).

Lobar microbleeds were not associated with OSAHS. We did not find any other significant association between several sleep parameters and OSAHS characteristics with SVD.

## 4. Discussion

To our knowledge, there are no studies in the Greek population that describe the neuroimaging characteristics of SVD associated with OSAHS. SVD, which is generally asymptomatic and commonly associated with cardiovascular risk factors, is a prevalent disease in older persons [18,19,20]. SVD has been studied extensively so far in Alzheimers’s disease and has been found to contribute to up to 45% of dementias [7,21]. The studies in OSAHS are limited but offer interesting results. In this study, the presence of SVD was evaluated with the use of the FS. In addition, we quantified for the first time B-WMH demonstrating a significant association in patients with OSAHS compared to controls.

These findings agree with previous studies [22,23,24] showing an association between SVD and OSAHS, suggesting also that vascular factors could be involved in the pathogenesis of SVD in patients. Lobar microbleeds were not associated with OSAHS [22,23].

We observed that patients with OSAHS had increased prevalence of cardiovascular risk factors when compared with controls. Wang et al. [25] have previously demonstrated that western diet, the metabolic syndrome, hypertension and obesity that are associated with changes in inflammation and microvessels have important roles in OSAHS, underlining the important correlation between the aforementioned factors and the development of SVD.

Similar results seem to emerge from the study of Chokesuwattanaskul et al., who observed an association between OSAHS and CSVD MRI findings of WMH and asymptomatic lacunar infarction (ALI) when compared to patients without OSAHS [22]. A recent meta-analysis supports that moderate to severe sleep apnea is positively related to WMH and silent brain infarction (SBI), which suggests that OSAHS probably plays an important role to the pathogenesis of CSVD [23]. In a study that investigated the possible relationship between OSAHS and cerebral SVD found that moderate-to-severe OSAHS is positively associated with multiple indicators of cerebral SVD, including WMH, cerebral microbleeds, and perivascular spaces. In addition, SVD was independently associated with increased AHI, particularly in patients with moderate-to-severe OSAHS [24].

The association between OSAHS and SVD may be bidirectional [26]. There aren’t many studies evaluating the complex relationship between OSAHS and SVD. One possible mechanism, according to the publications, is the activation of the systemic inflammation due to OSAHS, which results in the creation and the progression of SVD. According to some studies, C-Reactive Protein and Tumor Necrosis Factor are higher in the group of OSAHS [27,28]. Patients with OSAHS have persistently higher blood pressure which results in hypertension, which contributes to the development of SVD [29,30]. Patients with OSAHS have some cardiovasular risk factors which are involved in the pathophysiology of SVD [26]. Wang et al., showed that OSAHS results in endothelial dysfunction, increased levels of inflammatory biomarkers and a raise in arterial stiffness [31]. In patients with OSAHS, during sleep, hemodynamic changes including an increased intracranial pressure and an impaired endothelial function, can occur. The abnormal cerebrovascular function and the continuous episodes of hypoxia can result in a diffuse subcortical damage [26]. These changes are associated with SVD [32,33]. All things considered, the risk of SVD is possibly increased with one or more of the mechanisms above.

Our study is not without limitations. First, we acknowledge that we included a relatively small number of participants. Additionally, we did not assess intra- and interobserver reproducibility for brain imaging. Future longitudinal studies including larger cohort, may allow us to better clarify the potential association and causality—between SVD and OSAHS, preferably by including both histopathology samples and biomarkers. We acknowledge that the use of visual scales is useful when one aims to assess SVD on MRI. Early identification of SVD lesions could permit the application of possible preventive measures. Thus, we believe it is mandatory that all specialists that are involved in the care of OSAHS should be able to identify and classify SVD quantitatively.

## 5. Conclusions

In conclusion, our study demonstrated an independent significant association of OSAHS with SVD. In addition, the results confirmed for the first time the moderate association impact of OSAHS on B-WMH.

## Figures and Tables

**Table 1:** Sleep parameters and characteristics of OSAHS patients.

Sleep Parameters and Characteristics	OSAHS Patients (*n* = 24)
Age (years)	58 ± 11.9
Gender (Males)	15 (62.5%)
BMI (Kg/m^2^)	31.4 ± 6.2
ESS	9.9 ± 4.3
Sleep hours	7.3 ± 1.2
S1 (%)	4.9 ± 2.6
S2 (%)	63.3 ± 11.6
S3–4 (%)	10.1 ± 8.5
REM (%)	17.5 ± 28.4
AI (events/h)	16.3 ± 17.3
HI (events/h)	24.1 ± 13.4
AHI (events/h)	40.4 ± 21.1
ODI (events/h)	41.2 ± 23.2
MinSpO_2_ (%)	76.5 ± 12.8
T < 90% (min)	36.8 ± 65.6
Mean heart rate (beats/min)	68.1 ± 9.7

Data are expressed as mean ± SD or as frequencies (percentages). List of Abbreviations: BMI body mass index, ESS Epworth Sleepiness Scale, AHI apnea-hypopnea index, AΙ apnea, HΙ hypopnea, MinSpO_2_ minimum oxyhemoglobin saturation, ODI oxygen desaturation index, REM% rapid eye movement, S1% sleep stage 1, S2% sleep stage 2, S3–S4% sleep stage 3–4, T < 90% time with hemoglobin saturation < 90%.

**Table 2:** Vascular comorbidity of OSAHS patients and controls.

Vascular Diseases	OSAHS Patients (*n* = 24) N (%)	Controls (*n* = 34) N (%)
Hypertension	12 (50%)	7 (20.6%)
Coronary artery disease	4 (16.7%)	1 (2.9%)
Atrial fibrillation	2 (8.3%)	1 (2.9%)
Diabetes	4 (16.7%)	0 (0%)
Pulmonary Embolism	1 (4.2%)	0 (0%)
Dyslipidemia	14 (58.3%)	3 (8.8%)
Stroke	3 (12.5%)	0 (0%)
Peripheral arterial disease	1 (4.2%)	0 (0%)

**Table 3:** Comparison of patients with OSAHS and control group with respect to SVD in the multivariate analysis.

Independent Variable	OSAHS		Sig	aOR 95% CI	Adjusting (Univariate Analysis *p* < 0.1)
Dependent variable	DWM	Grade:0	ref		Age, gender, diabetes, dyslipidemia, hypertension, coronary artery disease, atrial fibrillation.
		Grade:1	0.033	8.66 (1.19–63.08)	
		Grade:2	0.862	-	
		Grade:3	0.999	-	
	PVWM	Grade:0	ref		Age, gender, dyslipidemia, hypertension, coronary artery disease, atrial fibrillation, valvular heart disease.
		Grade:1	0.002	104.98 (5.15–2141)	
		Grade:2	0.006	329.75 (5.32–204.38)	
	B-WMH	Yes/No	0.053	15.07 (0.97–234.65)	Age, gender, dyslipidemia, hypertension, coronary artery disease.

## Data Availability

The data that support the findings of this study are available on request from the corresponding author. The data are not publicly available due to containing information that could compromise the privacy of research participants.

## Supplementary Materials

The following are available online at https://www.mdpi.com/article/10.3390/diagnostics11091673/s1. Figure S1a: Coronary artery disease and presence of SVD in the deep white matter, Figure S1b: Diabetes and presence of SVD in the deep white matter, Figure S1c: Hypertension and presence of SVD in the deep white matter, Figure S1d: Duration of OSAS (months) and presence of SVD in the deep white matter, Figure S2a: Coronary artery disease and presence of SVD in the periventricular white matter, Figure S2b: Hypertension and presence of SVD in the periventricular white matter, Figure S2c: Duration of OSAS (months) and presence of SVD in the periventricular white matter.
