# Supplementary material for: Clinically Silent Small Vessel Disease of the Brain in Patients with Obstructive Sleep Apnea Hypopnea Syndrome
Source: Diagnostics (Basel). 2021 Sep 13;11(9):1673. doi: 10.3390/diagnostics11091673 (PMC8469951; doi:10.3390/diagnostics11091673)

Supplementary Material

Figure S1a: Coronary artery disease and presence of SVD in the deep white matter

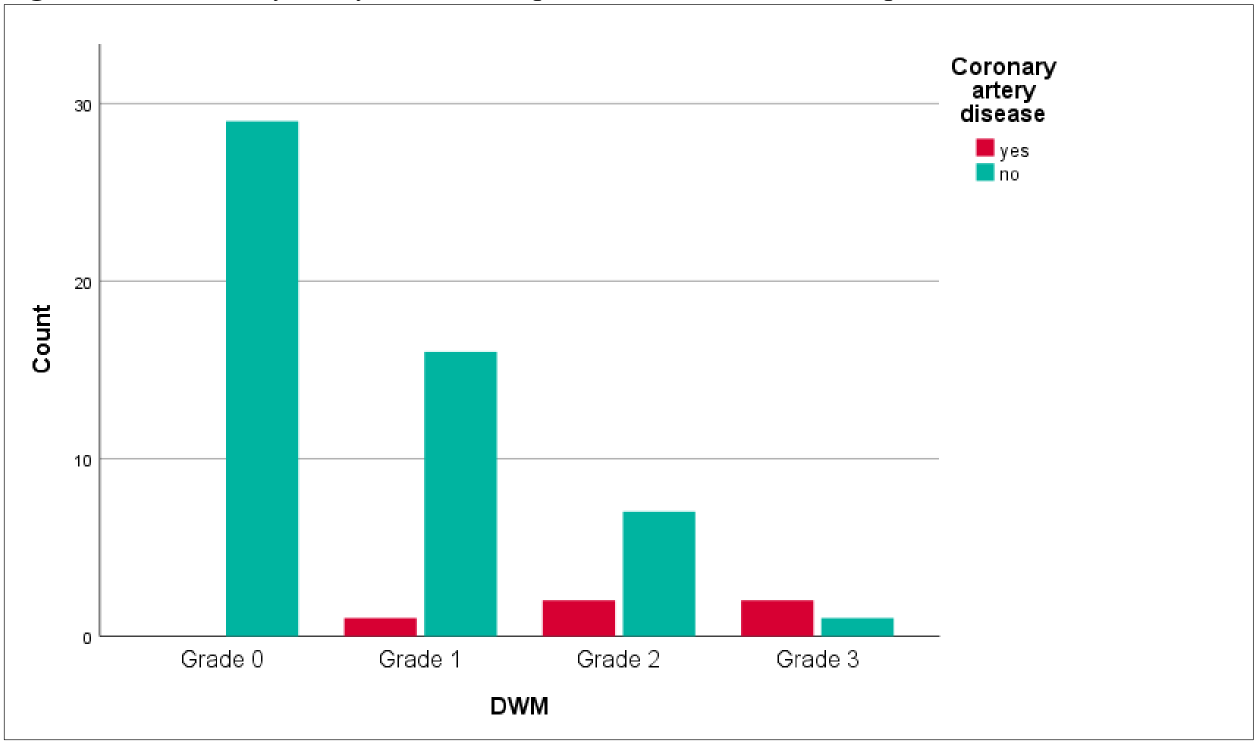

Figure S1b: Diabetes and presence of SVD in the deep white matter

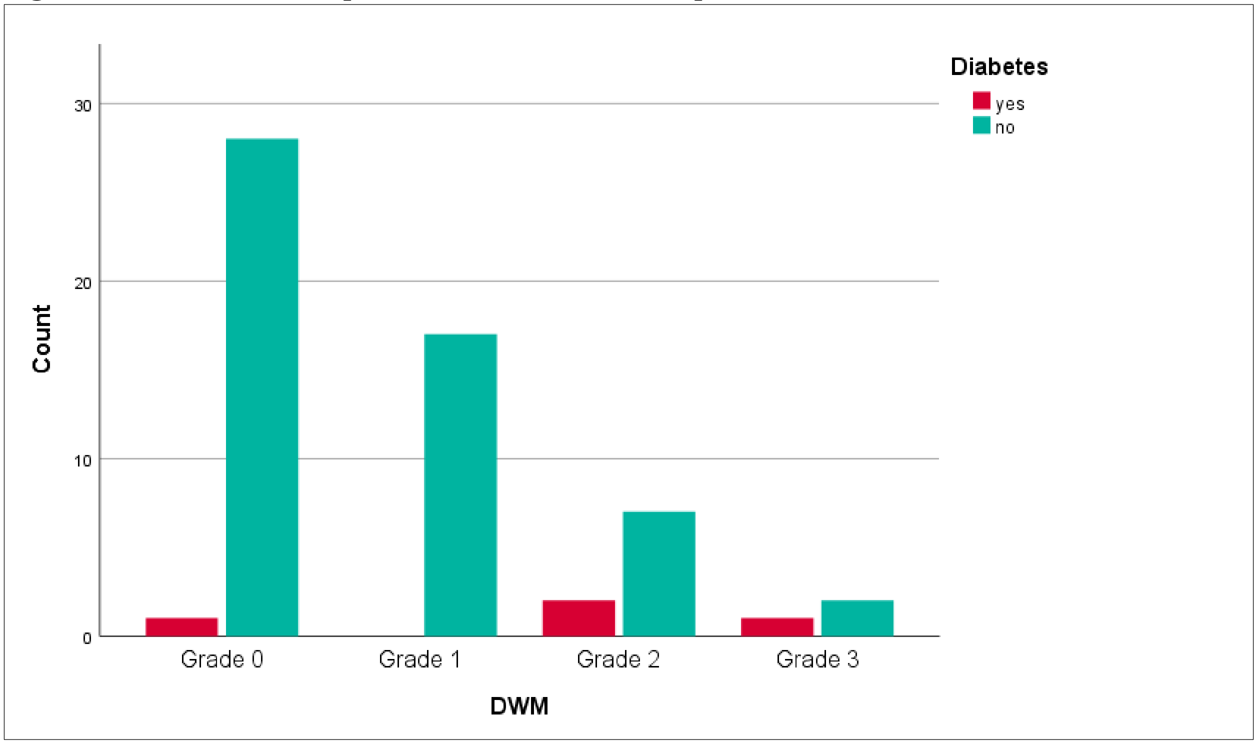

Figure S1c: Hypertension and presence of SVD in the deep white matter

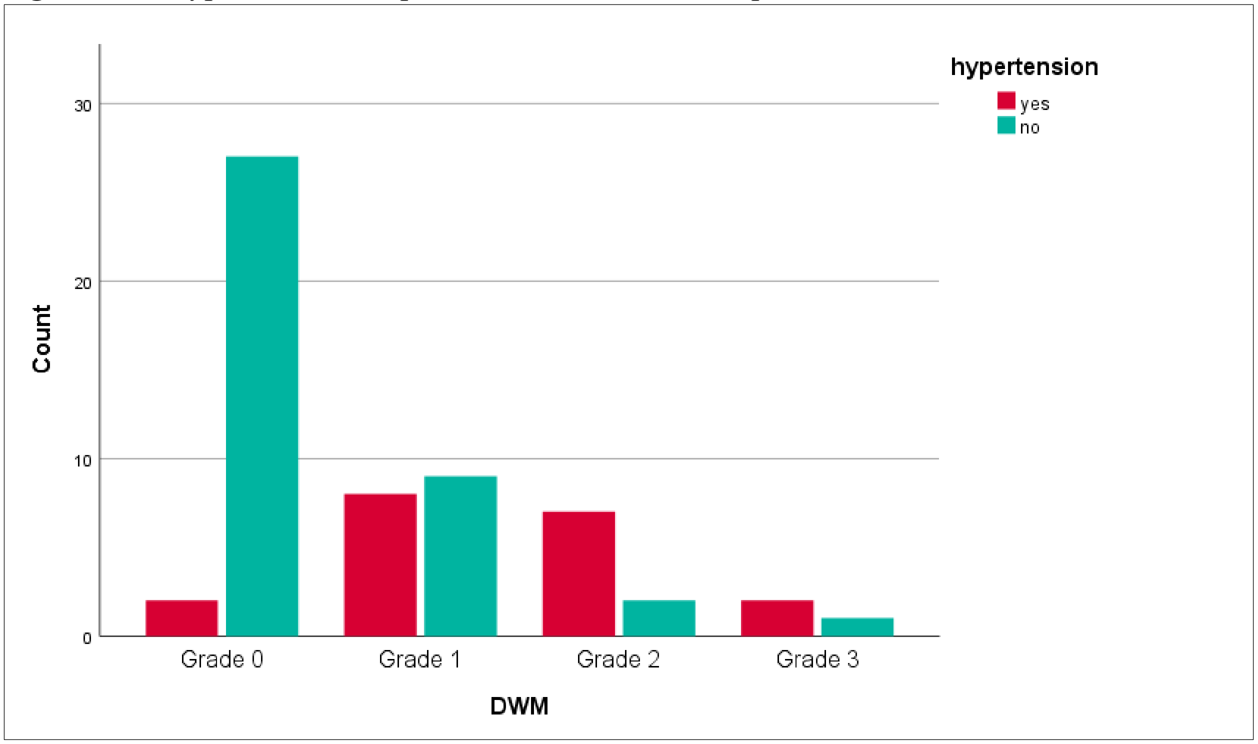

Figure S1d: Duration of OSAS (months) and presence of SVD in the deep white matter

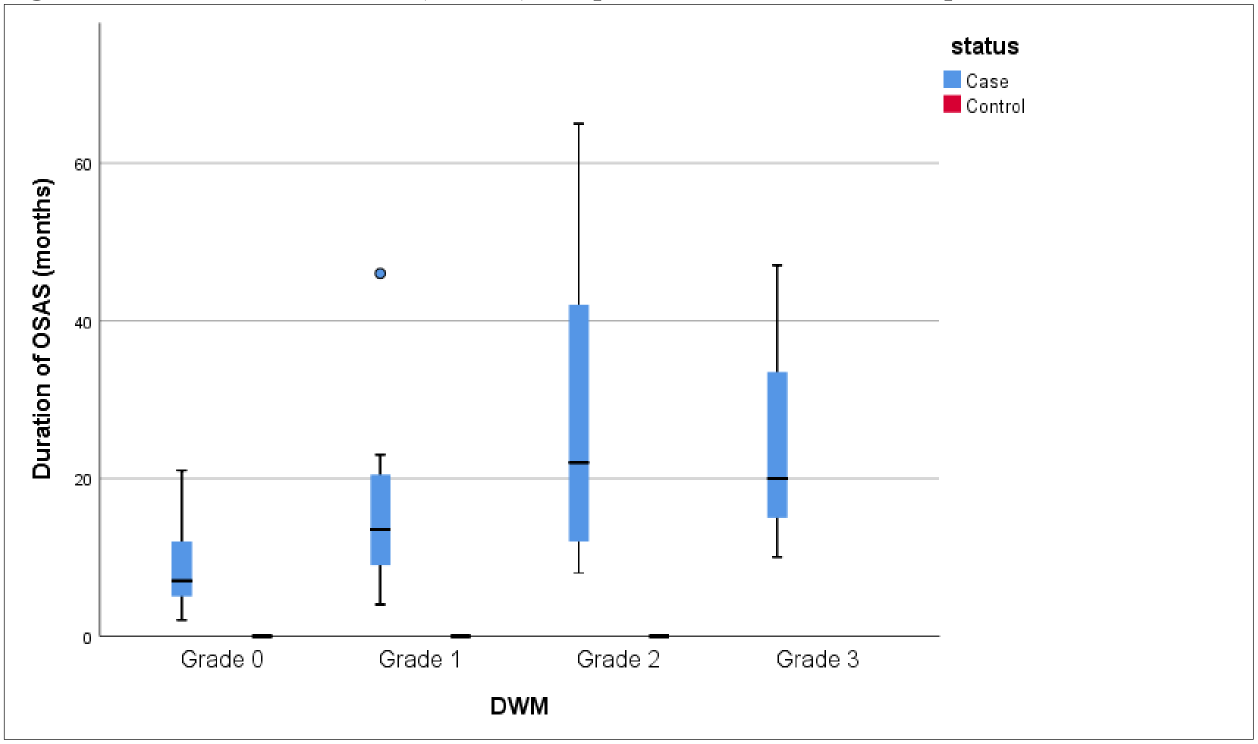

Figure S2a: Coronary artery disease and presence of SVD in the periventricular white matter

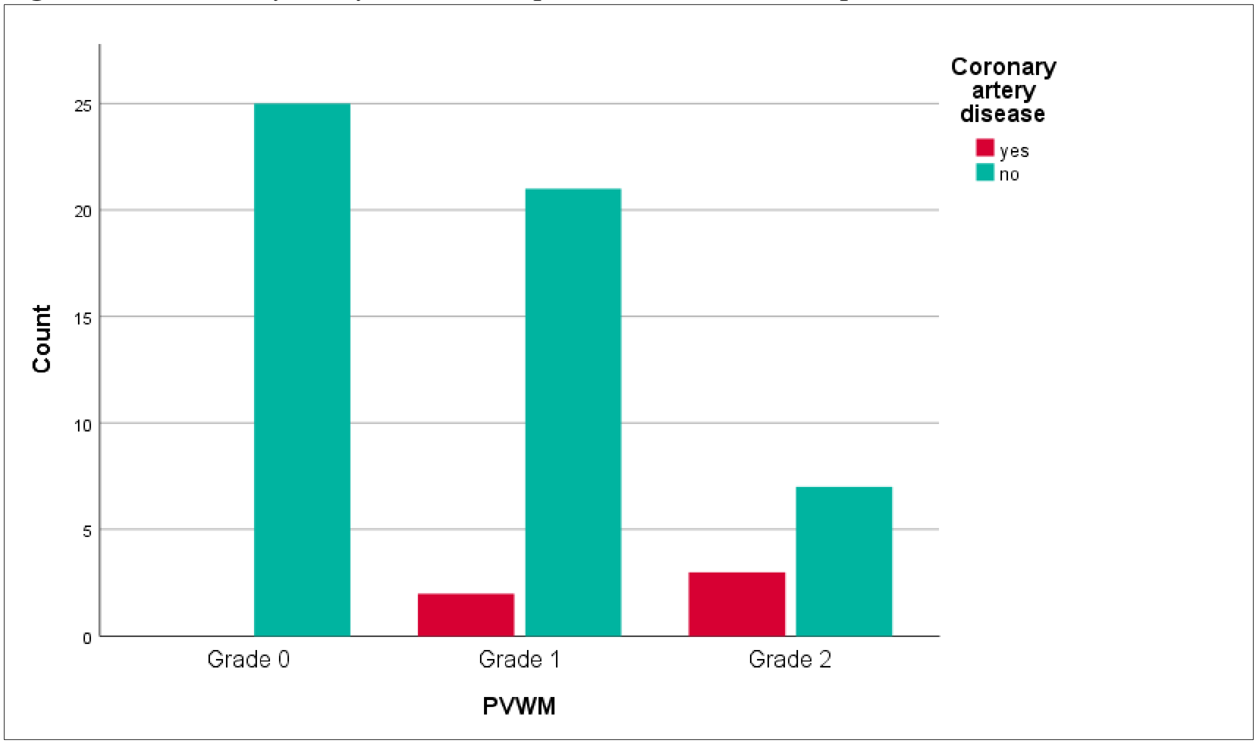

Figure S2b: Hypertension and presence of SVD in the periventricular white matter

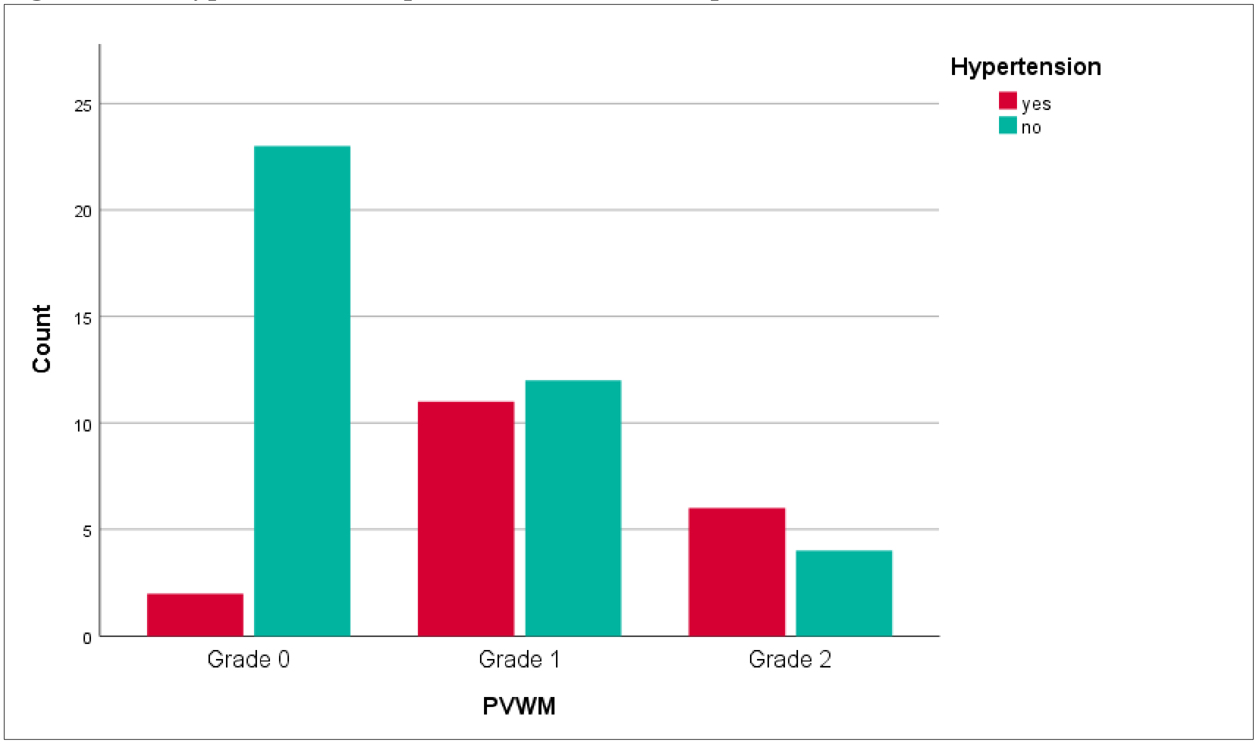

Figure S2c: Duration of OSAS (months) and presence of SVD in the periventricular white matter

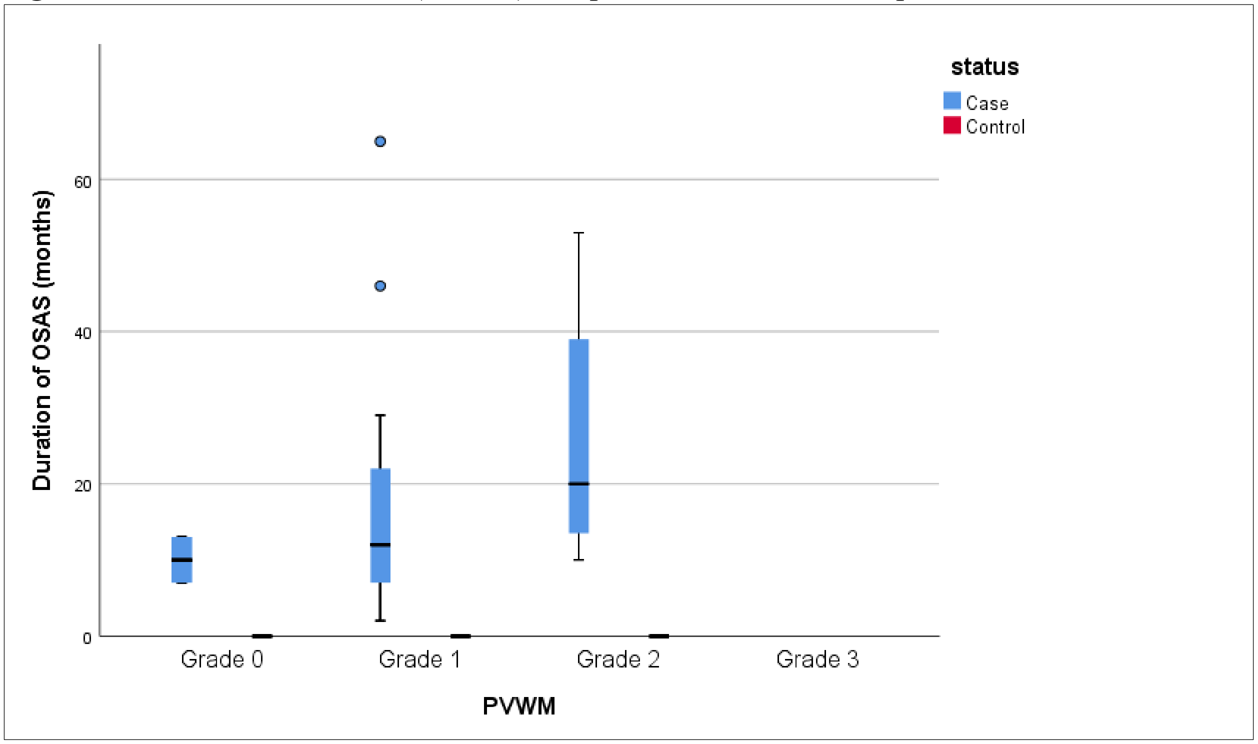

Supplement: Supplementary file 1 [file diagnostics-11-01673-s001.zip › diagnostics-1315731-supplementary.pdf]
